# Advanced atypical lung carcinoid tumour successfully treated with carboplatin, etoposide and atezolizumab: A case report

**DOI:** 10.1002/rcr2.951

**Published:** 2022-04-22

**Authors:** Yoshihiro Yamamoto, Kayo Ijichi, Ai Koike, Satoshi Nakamura, Yuriko Takahata, Yuki Okamatsu, Akitaka Fujita, Satoru Kawakami, Taishi Harada

**Affiliations:** ^1^ Department of Respiratory Medicine Japan Community Health Care Organization Kyushu Hospital Fukuoka Japan; ^2^ Department of Pathology Japan Community Health Care Organization Kyushu Hospital Fukuoka Japan

**Keywords:** atypical carcinoid tumour, case report, chemotherapy, immune checkpoint inhibitors, neuroendocrine tumour

## Abstract

Atypical carcinoid tumours are relatively rare among lung cancers. Surgery is regarded as standard treatment for localized cases, but there is little established evidence on treatment strategies for advanced cases. Moreover, the efficacy of immune checkpoint inhibitors for advanced carcinoid tumours is unclear. Here, we report a case of a patient with an atypical carcinoid tumour in whom successful disease control was achieved with the use of combined cytotoxic chemotherapy and immunotherapy.

## Graphical abstract

Immune checkpoint inhibitors are novel anticancer drugs increasingly used in the lung cancer field, except carcinoid tumour. We report a patient in whom an atypical carcinoid tumour was well controlled with platinum‐doublet chemotherapy and atezolizumab.

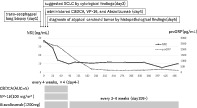

## INTRODUCTION

Atypical carcinoid tumours are a type of neuroendocrine tumour (NET). The neuroendocrine cells from which they derive are present in many organs, and thus NETs can arise in many different sites including the gastrointestinal tract, lung, thymus and ovary. After the gastrointestinal tract, the lung is the next most common site for NETs, accounting for 25% of all NETs. In addition, lung NETs occupy 1%–2% of all lung cancers.^1^ When NET lesions are localized, surgery is often regarded as the standard treatment, but in advanced cases there is some disagreement as to the optimum drug therapy. The immune checkpoint inhibitor (ICI), atezolizumab, is a breakthrough drug that has greatly expanded the range of treatments for lung cancer, either used alone or in combination with existing chemotherapy. However, ICIs are thought to be ineffective for lung carcinoid tumours because of very low intratumoural CD8^+^ cell infiltration and lack of programmed death‐ligand 1 (PD‐L1) expression—the target of atezolizumab. Nevertheless, trials are ongoing to evaluate immunotherapy in mixed populations of advanced NETs, including pulmonary carcinoids.^2^ Here, we describe a case of a patient with an atypical carcinoid tumour who had a good, sustained response to carboplatin, etoposide and atezolizumab.

## CASE REPORT

**FIGURE 1:**
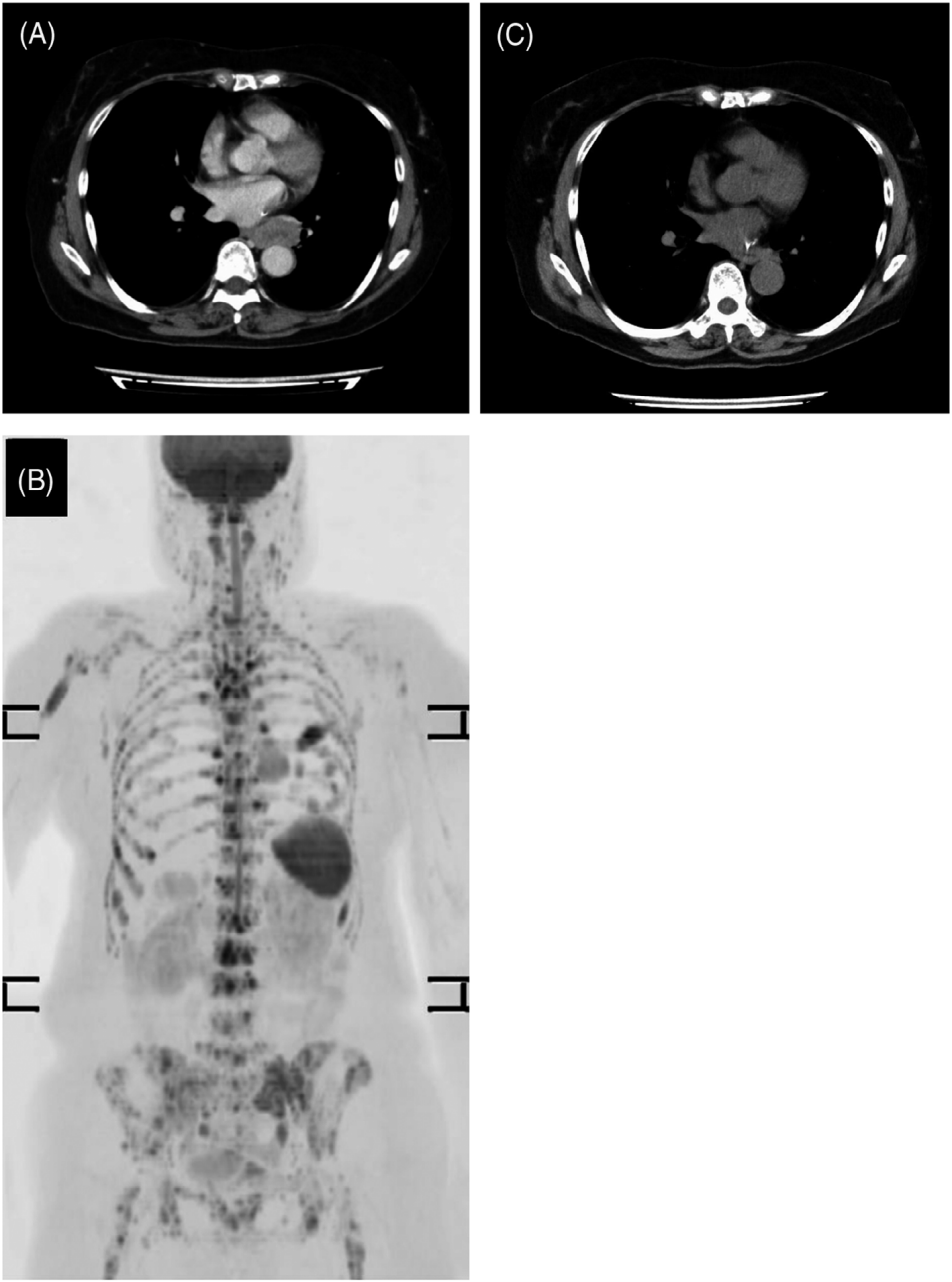
Chest contrast computed tomography (CT) at initial examination showing a substantial mass with slight enhancement in the lower lobe of the left lung (A). Whole‐body magnetic resonance diffusion‐weighted image showing systemic bone metastasis and lung tumour (B). Chest contrast CT 2 years after the initial examination showing shrinking of the tumour (C)

**FIGURE 2:**
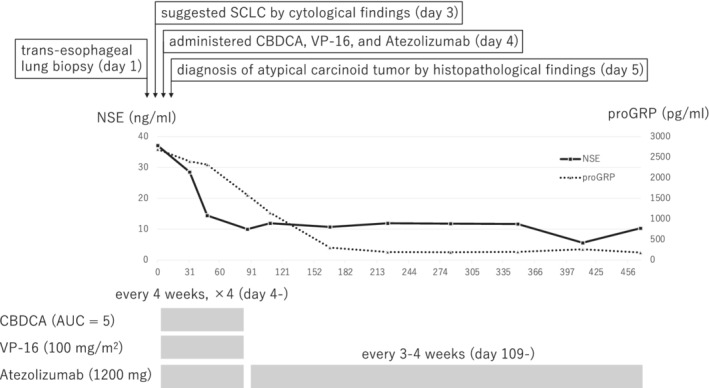
Clinical course of the patient throughout. CBDCA, carboplatin; NSE, neuron‐specific enolase; proGRP, pro‐gastrin‐releasing peptide, SCLC, small cell lung cancer; VP‐16, etoposide

**FIGURE 3:**
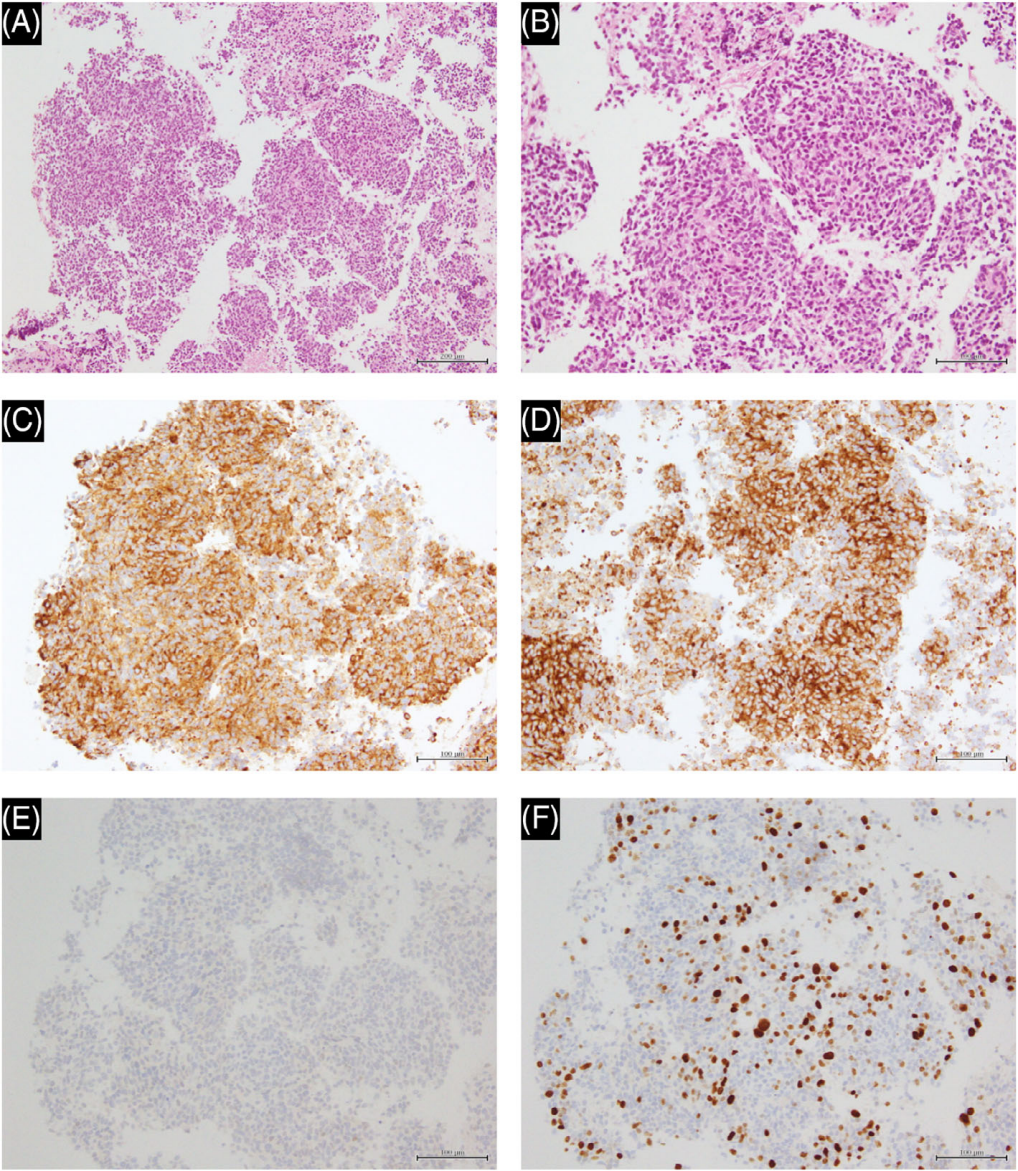
Atypical carcinoid tumour detected by trans‐oesophageal lung biopsy with haematoxylin–eosin stain. The tumour is growing in a sheeted or nested pattern. The tumour cells show high nucleus‐to‐cytoplasm ratio, and their nuclei show a round or oval shape and mild pleomorphism. One mitosis is present per 2 mm^2^. Necrosis is rarely seen (A, B). Chromogranin A (C) and synaptophysin (D) immunohistochemistry were strongly positive. Thyroid transcription factor‐1 was negative (E). The Ki‐67 labelling index was 20% (F)

A 63‐year‐old female patient was referred to our hospital because of back pain. Her Eastern Cooperative Oncology Group performance status was 0. Contrast computed tomography (CT) revealed a 4.2 × 2.1 × 5.0 cm substantial mass with slight enhancement in the lower lobe of the left lung and a hepatic mass, without lymphadenopathy (Figure 1A). Magnetic resonance imaging revealed extensive osteolytic disease from the lumbar spine to the cervical spine (Figure 1B). The tumour markers neuron‐specific enolase (NSE) and pro‐gastrin‐releasing peptide (proGRP) were elevated (37.1 ng/ml and 2690 pg/ml, respectively) (Figure 2). Trans‐oesophageal lung biopsy was performed. The cytological findings suggested small cell lung cancer (SCLC), so the patient was administered carboplatin (area under the plasma concentration vs. time curve: 5), etoposide (100 mg/m^2^) and atezolizumab (1200 mg) combination therapy. After the first cycle of the chemotherapy plus ICI therapy, the result of histopathological examination came out. It showed tumour cells arranged in sheeted or nested pattern, and scattered rosette‐like arrangements. There was no background necrosis, and mitosis was unremarkable. Immunohistochemically, the tumour cells were strongly positive for CD56, chromogranin A and synaptophysin, and negative for thyroid transcription factor‐1. Ki‐67 index was reported as 20% (223/1063) (Figure 3). The tumour was PD‐L1 negative. The tumour was diagnosed as atypical carcinoid based on the histopathological findings. Despite grade 4 neutropenia, which was successfully managed with Granulocyte Colony Stimulating Factor (G‐CSF), CT after two cycles showed slight shrinkage of lesions and that both NSE and proGRP were decreased (Figure 2). Therefore, the patient had continued carboplatin, etoposide and atezolizumab regimen for four cycles and then transferred to maintenance therapy with atezolizumab monotherapy (1200 mg). By the time the patient was moved to maintenance therapy, the lung tumour was seen to be shrinking over time on CT from 4.2 × 2.1 × 5.0 to 1.5 × 0.8 × 3.0 cm (Figure 1C), along with declining tumour marker levels (NSE 10.3 ng/ml and proGRP 183 pg/ml) (Figure 2). The hepatic mass and bone lesions had also shrunk over time. The patient is still in good general condition at 2 years after starting treatment and continues to be treated with atezolizumab monotherapy.

## DISCUSSION

Lung NETs are a heterogenous disease: at one end of the spectrum are the so‐called typical carcinoid tumours, which are well‐differentiated, low‐grade, slowly growing neoplasms, while at the other end of the spectrum are high‐grade neuroendocrine carcinomas, as typified by SCLC, which are aggressive and poorly differentiated. The primary treatment of localized lesions is surgery, while in advanced or unresectable cases systemic therapy, such as somatostatin analogues, peptide receptor radionuclide therapy, everolimus and chemotherapy, is the first‐line choice. In cases of rapid progression in particular, cisplatin‐ or carboplatin‐ based chemotherapy is often used with SCLC therapy, although cytotoxic regimens have demonstrated limited effects.^2^, ^3^ Data from a study of PD‐1 and PD‐L1 expression rates in 168 patients with pulmonary carcinoid tumours showed that high PD‐1 expression was detected in 16% of all carcinoid tumours. PD‐L1 expression was detected in 7% of typical carcinoid tumours; all atypical carcinoid tumours were PD‐L1 negative.^4^ However, in certain tumours, such as SCLC, PD‐L1 expression is not useful for predicting efficacy of ICIs. Large‐scale study demonstrates survival benefit of ICIs and cytotoxic chemotherapy combination therapy for patients with SCLC despite low expression of PD‐L1. Indeed, a few well‐managed cases of atypical pulmonary carcinoid treated with ICIs have been reported.^5^ However, to our knowledge, there have been no case reports of advanced atypical carcinoid tumours responding to cytotoxic chemotherapy and ICI combination therapy. At this time, there are no randomized controlled trials that have examined the effect of adding immunotherapy to chemotherapy for carcinoid tumours, but it is expected to be a promising treatment option in the future. Furthermore, the factors that differentiate treatment response are currently unclear. The accumulation of data on further cases may support ICIs as a standard treatment option for some atypical carcinoid tumours.

## CONFLICT OF INTEREST

None declared.

## ETHICS STATEMENT

Appropriate informed consent was obtained orally from the patient for publication of this case report and accompanying images. This study was approved by institutional review board.

## Data Availability

The data that support the findings of this study are available from the corresponding author upon reasonable request.
